# How Generative AI Use Styles Shape Academic Engagement: The Roles of Academic Impostor Syndrome and AI Policy Clarity

**DOI:** 10.3390/bs16060862

**Published:** 2026-05-27

**Authors:** Yu Wang, Xiaoxue Mi, Wenwen Tang, Yawei Tang, Heyuan Gao

**Affiliations:** 1 School of Business Administration, Dongbei University of Finance and Economics, Dalian 116025, China; 2025100151@stumail.dufe.edu.cn (X.M.);; 2 School of Urban Governance and Public Affairs, Suzhou City University, Suzhou 215000, China; tangwenwen@szcu.edu.cn; 3 Humanities and Social Sciences Department, Aviation University of Air Force, Changchun 130022, China; tangyw059@163.com

**Keywords:** AI use, AI interaction styles, academic impostor syndrome, academic engagement, AI policy clarity

## Abstract

Generative AI (GenAI) is increasingly embedded in higher education, yet evidence on its implications for students’ academic engagement and psychological experiences remains mixed. One possible reason is that prior research has often focused on how much students use AI and their general confidence in task completion, while paying less attention to how they use AI and how they attribute AI-supported achievement. To address this gap, this study distinguishes reflective from thoughtless AI use, examines academic impostor syndrome as a self-evaluative mechanism linking AI use styles to academic engagement, and tests perceived AI policy clarity as a contextual moderator. A two-wave survey of 478 Chinese university students showed that reflective AI use was negatively associated with academic impostor syndrome, whereas thoughtless AI use showed the opposite pattern. Academic impostor syndrome, in turn, was negatively associated with engagement and mediated both pathways. Perceived AI policy clarity amplified these patterns. These findings suggest that GenAI integration should be understood not only as a question of adoption or efficiency, but also of interaction quality and competence attribution. The study highlights the importance of cultivating reflective AI literacy and developing institutional policies that are clear yet psychologically attuned to students’ self-evaluative concerns.

## 1. Introduction

Generative artificial intelligence (GenAI) has quickly spread to higher education, and it is changing how students approach various academic tasks (e.g., writing, problem-solving, idea generation, coding, and exam preparation). Recent reviews indicate that GenAI has become an increasingly consequential part of students’ everyday academic work (Batista et al., 2024; Wu et al., 2025). In the Chinese higher education context, studies show that students’ GenAI use is shaped by academic competition, learning support needs, and institutional expectations. For example, Day (2025) found that Chinese postgraduate students may view AI as a resource for academic success and digitally mediated assistance, while also expressing concerns about over-reliance, diminished critical thinking, and an emerging “illusion of competence.” In transnational or English-medium learning environments, Chinese students may also position ChatGPT as a scaffold for writing, language mediation, and understanding course expectations, while still emphasizing verification and responsible use (Day, 2026).

At the same time, the growing presence of AI has intensified debates about academic integrity, fairness, overreliance, and the broader effects of reliance for students’ learning and development (Ocen et al., 2025; Sok & Heng, 2024; Wu et al., 2025). As a result, the central question is no longer whether students use AI, but how they use it when completing important academic tasks. Emerging studies suggest that students’ use of AI is not uniform. Research on reliance on GenAI, for example, shows patterns such as reflective, cautious, collaborative, and thoughtless use (Hou et al., 2025a, 2025b). Similarly, studies of students’ learning approaches with GenAI indicate that AI-supported activity can range from reflective and resourceful engagement to shortcut-oriented and passive use (Yang et al., 2024). These findings suggest that a focus only on usage frequency or general dependence may miss more meaningful differences in how students engage with AI.

To better capture these differences, the present study draws on the distinction between augmentation-oriented and automation-oriented human–AI collaboration (Raisch & Krakowski, 2021). In general, augmentation refers to uses of AI that support and extend human judgment, whereas automation refers to uses of AI that substitute for human effort. Augmentation-oriented AI use typically involves authorial control, active selection, metacognitive engagement, and epistemic caution. In contrast, automation-oriented AI use is more closely related to cognitive offloading, passive uptake, and unreflective acceptance (Perdana et al., 2026). On this basis, the present study focuses on two representative AI use styles in core academic tasks: reflective AI use and thoughtless AI use. Rather than treating these two styles as opposite ends of a single continuum, they are conceptualized as distinct tendencies that potentially coexist within the same individual (Hou et al., 2025b).

Distinguishing between these AI use styles is important because they may shape students’ academic engagement in different ways. Academic engagement is a core marker of students’ learning quality and overall developmental functioning. It is a multidimensional construct encompassing behavioral, cognitive and emotional engagement, and is strongly related to students’ learning attention, academic achievement, and emotional well-being (Heung & Chiu, 2025). While AI-assisted learning may strengthen student engagement, overusing AI tools could also reduce critical thinking, increase disengagement, and lower participation in meaningful learning (Heung & Chiu, 2025; Lo et al., 2024). Recent empirical evidence further suggests that AI tool use may be associated with lower critical thinking through epistemic laziness and metacognitive weakness, highlighting the risk that students may rely on AI outputs without sufficient inquiry or self-monitoring (Yurt & Kuşci, 2026). Such mixed findings underline that it is necessary to distinguish different AI use patterns, rather than simply regarding AI use as positive or negative.

To explain why AI use styles may relate to engagement, the present study focuses on academic impostor syndrome as a self-evaluative mechanism. In AI-assisted academic contexts, students may feel capable of completing a task with AI support while still questioning whether the outcome genuinely reflects their own effort, judgment, and competence. Prior research suggests that uncritical reliance on AI may heighten authenticity and ownership concerns, whereas more agentic and reflective approaches may help students maintain psychological ownership over their work (Batista-Toledo & Gavilan, 2026; Domingo, 2025; Tsao, 2025; Vassallo, 2026).

Importantly, the meaning and the outcomes of the use of AI cannot be separated from the institutional context. As GenAI is becoming more integrated into higher education, universities are under pressure to provide guidance not only on academic integrity but also on pedagogy, accountability, training, and responsible AI use (Chan, 2023). However, the effect of such policies depends less on their formal existence and more on whether students perceive them as clear and practically feasible. Recent evidence shows that ambiguity in institutional AI policies places greater interpretive demands on students and instructors, creating more uncertainty and concerns about fairness, while clearer guidance provides a more consistent basis for judging the legitimacy of AI-assisted academic work (Tang et al., 2025; Tsao, 2025). Therefore, the present study treats AI policy clarity as a critical boundary condition that may shape how students interpret the implications of their own AI use for competence, ownership, and legitimacy.

Against this background, the present study develops and tests a moderated mediation model linking students’ AI use styles in core academic tasks to academic engagement through academic impostor syndrome, with AI policy clarity as a first-stage moderator. This study extends existing AI-assisted learning research in three ways. First, it moves beyond general measures of AI usage frequency by distinguishing between reflective and thoughtless AI use. Second, it shifts the focus from task-completion confidence to a deeper question: whether students experience AI-supported achievements as authentic, deserved, and attributable to their own competence. Third, it highlights AI policy clarity as an important contextual moderator, showing how institutional guidance may shape not only students’ behavior but also how they interpret and internalize AI-supported achievement. Together, these contributions offer a more psychologically informed and educationally grounded understanding of how GenAI influences student learning in higher education.

## 2. Literature Review and Hypothesis Development

### 2.1. Reflective and Thoughtless AI Use in Core Academic Tasks and Academic Impostor Syndrome

Although students use generative AI in many educational situations, the meaning and consequences of such use are unlikely to be the same across task contexts. The present study focuses on students’ AI use in core academic tasks because these tasks are more directly tied to authorship, the display of competence, academic accountability, and evaluative pressure. Prior review studies show that students increasingly use GenAI for writing, problem-solving, idea generation, coding, and exam preparation. At the same time, concerns appear to vary substantially depending on how AI is used in these academic contexts, rather than on use itself (Batista et al., 2024; Tang et al., 2025; Wu et al., 2025).

Building on the automation–augmentation distinction, this section further specifies reflective AI use and thoughtless AI use in the context of core academic tasks. Reflective AI use refers to an augmentation-oriented pattern, in which students critically evaluate, revise, and purposefully integrate AI-generated outputs while retaining judgment and authorial control. In contrast, thoughtless AI use refers to a more automation-oriented pattern, where students delegate substantial cognitive work to AI, adopt AI-generated outputs with limited scrutiny, and show lower cognitive engagement in task completion. These styles are not treated as opposite ends of a continuum, but as analytically distinct tendencies that may coexist to varying degrees in students’ academic behaviors. This treatment is consistent with recent scale-development research showing that reflective use and thoughtless use constitute two separate dimensions of AI reliance rather than direct opposites (Hou et al., 2025b).

The automation–augmentation distinction explains why reflective and thoughtless AI use represent qualitatively different interaction styles, whereas self-determination theory clarifies why these styles may have different implications for students’ perceived autonomy, competence, and ownership. According to self-determination theory, the satisfaction of basic psychological needs for autonomy and competence is essential for an individual’s internal sense of self-worth and optimal functioning (Deci & Ryan, 2000; Ryan & Deci, 2000). From this perspective, reflective and thoughtless AI use should differ in how they support or undermine students’ autonomy, competence, and ownership during core academic tasks. Reflective AI use may help students maintain active evaluation, ongoing monitoring, and a sense of ownership over task completion, thereby supporting self-authorship and perceived competence. By contrast, thoughtless AI use may shift cognitive processing to AI systems, weaken students’ sense of ownership, and make academic outcomes feel less reflective of their own capabilities (Neufeld et al., 2023). Existing empirical findings offer general support for this conceptual distinction. Reflective and thoughtful forms of AI reliance are associated with critical engagement, metacognitive monitoring, and student agency, whereas thoughtless or over-reliant use is more closely tied to passive adoption, reduced scrutiny, and displaced cognitive effort (Hou et al., 2025a; Marín Díaz, 2025; Yang et al., 2024). Experimental evidence further suggests that low-agency AI use can diminish students’ psychological ownership over their work, whereas more agentic and effective AI use is associated with lower impostor-like feelings over time (Batista-Toledo & Gavilan, 2026). Educational work on automation and augmentation also indicates that what appears to be AI-enabled empowerment may sometimes conceal a more troubling dynamic of cognitive outsourcing, in which students conflate AI fluency with genuine understanding (Perdana et al., 2026).

These differences are especially relevant to academic impostor syndrome. Academic impostor syndrome refers to a self-evaluative experience in which individuals perceive themselves as less competent than others believe them to be and tend to attribute success to external factors rather than to their own ability (Y. Wang & Li, 2023). Rather than reflecting only low confidence, impostor feelings involve a deeper sense that one’s apparent success may not genuinely represent one’s own competence, often accompanied by fear of being exposed as undeserving or inadequate (Y. Wang & Li, 2023). In this sense, academic impostor syndrome is conceptually distinct from general academic self-efficacy, academic insecurity, or academic anxiety, because it centers on the perceived authenticity and deservedness of one’s achievements rather than only on perceived capability, general self-doubt, or emotional distress. Such experiences might become particularly salient in the context of AI-assisted academic work, when students perceive their performance as more dependent on external technological support rather than on their own effort and judgment. Emerging evidence suggests that heavy reliance on AI tools may heighten concerns about authenticity, self-worth, and whether academic success truly belongs to the student rather than to the tool (Domingo, 2025).

Taken together, these arguments suggest that reflective AI use in core academic tasks may reduce academic impostor syndrome by preserving autonomy, competence, and ownership over academic work. Thoughtless AI use, by contrast, may heighten academic impostor syndrome by weakening students’ sense of authentic competence and increasing external attribution of success. Accordingly, the following hypotheses are proposed:

**H1a.**  *Reflective AI use in core academic tasks is negatively associated with academic impostor syndrome.*

**H1b.**  *Thoughtless AI use in core academic tasks is positively associated with academic impostor syndrome.*

### 2.2. The Mediating Role of Academic Impostor Syndrome

Academic impostor syndrome provides a useful lens for understanding why different AI use styles may be associated with academic engagement. Prior studies on AI-assisted learning have often relied on academic self-efficacy to explain the motivational benefits of AI use (Almohammadi et al., 2025; Zhang & Xu, 2025). These mechanisms remain important because GenAI can help students complete academic tasks more efficiently and may increase their perceived capability. However, in GenAI-supported academic work, confidence in task completion may become psychologically ambiguous. Students’ perceived capability may reflect not only their own competence, but also their ability to use AI effectively and the support provided by the tool itself. Recent evidence also shows that GenAI use may increase students’ confidence and task efficiency while also intensifying technological dependence (Zhang & Xu, 2025). Thus, confidence in AI-assisted task completion does not necessarily indicate a secure sense of personal competence. This paradox is especially important for the present study because it suggests that students’ confidence in completing AI-assisted tasks may not always indicate a secure sense of personal competence. Rather, such confidence may coexist with dependence, cognitive offloading, and uncertainty about whether the final performance genuinely reflects the student’s own mastery (Zhang & Xu, 2025).

This ambiguity makes academic impostor syndrome a theoretically appropriate mediator. Unlike general academic self-efficacy, insecurity, or anxiety, academic impostor syndrome concerns whether achievements are experienced as authentic, deserved, and attributable to one’s own competence (Todd & Mcilroy, 2025; Y. Wang & Li, 2023). In GenAI-supported learning, students may be able to produce acceptable academic outcomes while still questioning whether those outcomes genuinely reflect their own effort, judgment, and ability. This concern is consistent with emerging evidence linking AI use to issues of authenticity, academic legitimacy, guilt, and psychological ownership (Batista-Toledo & Gavilan, 2026; Domingo, 2025; Qu & Wang, 2025; Vassallo, 2026). Thus, academic impostor syndrome shifts attention from task-completion confidence to the process through which students attribute and internalize AI-assisted achievement.

Such self-evaluative vulnerability is likely to undermine academic engagement. Students with stronger impostor feelings may find it more difficult to experience their academic efforts and achievements as authentic expressions of their own competence (Neufeld et al., 2023). From a self-determination theory perspective, these feelings signal a fragile sense of competence and weakened ownership, both of which may erode students’ behavioral, cognitive, and emotional investment in learning (Reeve, 2012). When students doubt the legitimacy of their own performance, they may become less willing to participate actively, invest cognitive effort, or feel emotionally connected to academic tasks. This reasoning is also consistent with review evidence suggesting that AI-supported learning can foster engagement, whereas disengagement may emerge when students become over-reliant on AI or show reduced critical involvement in learning (Heung & Chiu, 2025; Lo et al., 2024).

Accordingly, reflective AI use is expected to support academic engagement indirectly by reducing students’ vulnerability to academic impostor syndrome, whereas thoughtless AI use is expected to undermine engagement indirectly by heightening such feelings. Therefore, the following hypotheses are proposed:

**H2a.**  *Academic impostor syndrome mediates the positive relationship between reflective AI use in core academic tasks and academic engagement.*

**H2b.**  *Academic impostor syndrome mediates the negative relationship between thoughtless AI use in core academic tasks and academic engagement.*

### 2.3. The Moderating Role of AI Policy Clarity

As generative AI becomes more embedded in higher education, institutional policies are increasingly expected to offer guidance not only on academic integrity, but also on pedagogy, accountability, training, and responsible use (Chan, 2023). However, the implications of such policies depend not simply on their formal existence, but on whether students perceive the boundaries of acceptable AI use as clear and understandable. In the present study, AI policy clarity is defined as students’ perceived clarity regarding institutional guidelines for GenAI use, anticipated instructor responses to inappropriate use, and the boundaries of academically acceptable practice (Tang et al., 2025). This conceptualization allows us to treat policy clarity as an individual-level interpretive condition rather than as a purely macro-level institutional characteristic.

Based on Social Information Processing Theory, individuals rely on environmental cues to decide which behaviors are appropriate, acceptable, or risky in a particular situation (Ramirez et al., 2002; Tidwell & Walther, 2002). In academic settings, institutional policies can serve as such cues by signaling the normative boundaries of acceptable AI use. Ambiguity in institutional AI policy can shift the interpretive burden to students and teachers, creating uncertainty, concerns about fairness, and anxiety about inadvertently crossing ethical boundaries (Tsao, 2025). In contrast, clearer policy messaging and more explicit guidance may give students a more stable and concrete basis for judging the legitimacy of their own AI-assisted academic behavior (Chan, 2023; Tsao, 2025). Put differently, policy clarity may shape not only what students believe they are allowed to do, but also how students attribute competence and authorship in AI-assisted academic work.

This interpretive function of policy clarity suggests that it may reinforce the divergent psychological implications of reflective and thoughtless AI use. When institutional expectations are clear, students may be more likely to recognize that thoughtless or over-reliant AI use departs from legitimate academic participation and weakens the extent to which task outcomes can be experienced as authentically their own. Under such conditions, thoughtless AI use may show a stronger positive association with academic impostor syndrome. By contrast, when the boundaries of acceptable AI use are clear, reflective AI use may be more readily understood as a responsible and academically legitimate form of tool-supported learning. In turn, such clarity may strengthen the extent to which reflective use supports students’ sense of agency, ownership, and authentic competence, thereby reinforcing its protective effect against academic impostor syndrome. This line of reasoning corresponds to the empirical evidence that clear regulations, transparent protocols, and available guidance are essential to reduce uncertainty and promote the responsible use of AI in higher education (Chan, 2023; Tang et al., 2025).

Taken together, these arguments suggest that perceived AI policy clarity does not simply exert a direct influence on students’ impostor feelings. Rather, it shapes how students interpret the legitimacy and competence implications of their own AI use in core academic tasks. Accordingly, policy clarity is expected to amplify the divergent effects of reflective and thoughtless AI use on academic impostor syndrome. Therefore, the following hypotheses are proposed:

**H3a.**  *AI policy clarity moderates the positive relationship between thoughtless AI use in core academic tasks and academic impostor syndrome, such that this relationship is stronger when perceived AI policy clarity is high.*

**H3b.**  *AI policy clarity moderates the negative relationship between reflective AI use in core academic tasks and academic impostor syndrome, such that this relationship is stronger when perceived AI policy clarity is high.*

### 2.4. Conditional Indirect Effects of AI Use Styles on Academic Engagement

The preceding arguments suggest that the effects of students’ AI use styles on academic engagement are unlikely to be uniform across institutional conditions. Reflective and thoughtless AI use are expected to differentially influence academic impostor syndrome, which in turn is associated with students’ behavioral, cognitive, and emotional engagement in core academic tasks. At the same time, the strength of the relationship between AI use style and academic impostor syndrome is expected to depend on students’ perceptions of AI policy clarity. Therefore, the indirect effects of AI use styles on academic engagement via academic impostor syndrome are not fixed but contingent on how clearly students perceive institutional AI policies and expectations. This integrated framework is consistent with the perspective that students’ AI-assisted performance results from an interaction between their individual use patterns and the institutional cues that shape the interpretation of that use (Tang et al., 2025; Tsao, 2025).

More specifically, when AI policy clarity is high, reflective AI use is more likely to be associated with reduced academic impostor syndrome and, consequently, greater academic engagement. Conversely, under high policy clarity, thoughtless AI use is more likely to be linked to increased academic impostor syndrome and, in turn, lower academic engagement. Therefore, AI policy clarity is expected to moderate the indirect effects of reflective and thoughtless AI use on academic engagement via academic impostor syndrome. The following hypothesis is thus proposed:

**H4a.**  *AI policy clarity moderates the indirect association between reflective AI use and academic engagement through academic impostor syndrome, such that this positive indirect association is stronger when perceived AI policy clarity is high.*

**H4b.**  *AI policy clarity moderates the indirect association between thoughtless AI use and academic engagement through academic impostor syndrome, such that this negative indirect association is stronger when perceived AI policy clarity is high.*

**Figure 1:**
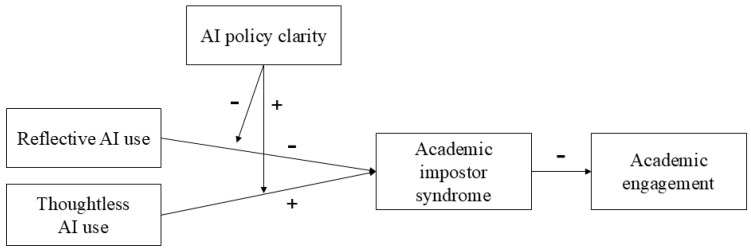
Theoretical model.

The proposed moderated mediation model is summarized in Figure 1.

## 3. Materials and Methods

### 3.1. Participants and Procedure

As temporal separation between predictor and criterion measures has been recommended as a procedural remedy for reducing common method variance concerns (Podsakoff et al., 2003), this study adopted a two-wave survey design. Although prior methodological work has emphasized that no universally optimal time lag exists, the appropriate interval should reflect the nature of the constructs and the rhythm of the research context (Dormann & Griffin, 2015). Intervals of approximately one month have been commonly used in educational research examining engagement and related psychological experiences (Salmela-Aro & Upadyaya, 2014). Accordingly, Time 1 (T1) data were collected between 10 October and 20 October 2025, and Time 2 (T2) data were collected one month later, between 10 November and 20 November 2025. T1 data collection was scheduled approximately one month after the beginning of the semester, when students had already engaged in their courses for several weeks and their GenAI use patterns in academic tasks were likely to be relatively stable. T2 data collection was scheduled approximately one month later, between the midterm and final examination periods, allowing us to examine subsequent associations while reducing potential interference from end-of-semester examination pressure. At T1, participants reported on reflective AI use, thoughtless AI use, AI policy clarity, and the control variables. At T2, the same participants completed the measures of academic impostor syndrome and academic engagement.

Participants were recruited through online questionnaires using a snowball sampling strategy. To anonymously match responses across the two survey waves, participants were asked to generate a self-generated four-digit code; this code was used only to link each participant’s T1 and T2 responses and could not be used to identify participants personally. Participation was entirely voluntary. Before each survey wave, participants were presented with an informed consent form explaining the purpose of the study, the confidentiality of the data, the voluntary nature of participation, and their right to withdraw without any negative consequences. No personally identifiable information was collected at any stage of the research. The data were used only for academic research and are reported in aggregate form.

A total of 556 responses were obtained at T1. After cross-wave matching and the removal of invalid cases (i.e., failed attention checks, incomplete or missing matching codes, and unmatched responses), the final analytical sample was 478. The demographic and academic characteristics are provided in Table 1. Attrition analyses were conducted to compare participants who completed both waves and were included in the final sample (*N* = 478) with those who completed Time 1 but were not retained in the final matched sample (*N* = 78). Independent-samples t-tests showed no significant differences between the two groups in AI use frequency, reflective AI use, thoughtless AI use, AI policy clarity, or self-reported academic performance, *ps* > 0.05. Chi-square tests also indicated no significant differences in major, *χ*^2^(4) = 5.94, *p* = 0.204, or academic year, *χ*^2^(3) = 0.47, *p* = 0.925. However, the two groups differed significantly in gender distribution, *χ*^2^(1) = 4.07, *p* = 0.044, with female students being more represented in the retained sample. Gender was therefore retained as a control variable in all hypothesis-testing analyses.

### 3.2. Measures

#### 3.2.1. Reflective and Thoughtless AI Use

Reflective and thoughtless AI use were measured at Time 1 using subscales adapted from Hou et al. (2025b). Before responding to these items, participants were instructed to recall their use of generative AI tools (e.g., ChatGPT, DeepSeek) when completing core academic tasks since the start of the current semester. Core academic tasks were defined as learning activities closely related to students’ major courses, including course assignments, literature review, paper writing, problem solving, data processing and analysis, programming or modeling. We focused on core academic tasks because these activities are directly tied to academic evaluation, competence demonstration, and students’ sense of authorship over their work.

The original scale was developed to assess undergraduate students’ AI use behavior in problem-solving activities. In the present study, the items were adapted to the context of core academic tasks by revising the item stems accordingly. Reflective AI use was measured with four items. A sample item is “When completing core academic tasks, I read Generative AI’s output critically when the output was generated.” Thoughtless AI use was also measured with four items. A sample item is “When completing core academic tasks, I adopted Generative AI’s output without major changes.” Responses were rated on a five-point Likert scale (1 = almost never, 5 = almost always). Cronbach’s alpha values were 0.840 for reflective AI use and 0.815 for thoughtless AI use.

#### 3.2.2. Academic Impostor Syndrome

Academic impostor syndrome was assessed at Time 2 with a 10-item scale developed by Todd and Mcilroy (2025). This scale measures individuals’ tendency to doubt the authenticity of their academic ability. Sample items include “I am not as clever as many other students on my course” and “I believe that when I do well, it is by accident.” Responses were rated on a 7-point Likert scale (1 = strongly disagree, 7 = strongly agree). In the present study, the scale showed high internal consistency (α = 0.934).

#### 3.2.3. AI Policy Clarity

AI policy clarity was measured at Time 1 using four items adapted from the clarity dimension of organizational transparency developed by Schnackenberg et al. (2021). This dimension was selected because the present study focused on students’ perceived lucidity and comprehensibility of institutional AI-use guidance, rather than on the amount or factual accuracy of policy information. The items were contextualized to the higher education setting by replacing the original organizational referent with the university’s AI use policy. A sample item is “My university’s AI use policy is clear.” Participants responded on a 5-point Likert scale (1 = strongly disagree, 5 = strongly agree). In this sample, Cronbach’s alpha was 0.878.

#### 3.2.4. Academic Engagement

Academic engagement was measured at Time 2 using the University Student Engagement Inventory (USEI). The USEI conceptualizes student engagement as a multidimensional construct consisting of behavioral, emotional, and cognitive engagement. The scale includes 15 items, with five items for each dimension. Sample items include “I usually participate actively in group assignments” for behavioral engagement, “I am interested in the school work” for emotional engagement, and “I try to integrate the acquired knowledge in solving new problems” for cognitive engagement. Items were rated on a 5-point Likert scale (1 = never, 5 = always). Prior research has supported the factorial and cross-cultural validity of the USEI across multiple regions and language versions (Assunção et al., 2020). In the present study, the scale demonstrated good internal consistency (Cronbach’s α = 0.812).

#### 3.2.5. Control Variables

Gender, age, academic year, major, self-reported academic performance, and AI use frequency were included as control variables because prior research suggests that these demographic and academic characteristics may be associated with GenAI use, AI reliance, academic self-efficacy, and engagement-related experiences (Jia et al., 2025; Wu et al., 2025; Zhang & Xu, 2025). Academic performance was controlled because prior achievement may shape students’ perceived competence and academic engagement (Jia et al., 2025; Zhang & Xu, 2025). AI use frequency was controlled to distinguish AI use styles from the general intensity of AI exposure, which has been linked to AI reliance, perceived efficacy, and technology-related learning experiences (Wu et al., 2025; Zhang & Xu, 2025). In this study, gender was coded as 0 = male and 1 = female. Academic year was coded as 1 = first-year, 2 = second-year, 3 = third-year, and 4 = fourth-year or graduating-year student. Major was coded as 1 = humanities and social sciences, 2 = science, 3 = engineering, 4 = agriculture, and 5 = medicine. Self-reported academic performance was assessed by asking participants to indicate their overall academic ranking relative to students in the same major or academic year. The original response options ranged from 1 = top 10% to 5 = bottom 25%; this variable was reverse-coded before analysis so that higher scores indicated better self-reported academic ranking. AI use frequency was measured with three items assessing how often students used AI tools for (1) computational tasks, including programming, mathematical problem-solving, and data analysis; (2) information retrieval and literature review; and (3) writing and creative generation. Responses were recorded on a 6-point scale ranging from 1 (never) to 6 (multiple times daily). The mean score of the three items was used as the AI use frequency score, with higher scores indicating more frequent AI use in academic tasks. All control variables were measured at Time 1.

### 3.3. Analytic Strategy

Data analysis was conducted using IBM SPSS 26.0, Mplus 8.0, and the PROCESS macro for SPSS, version 4.0. Harman’s single-factor test was used to assess potential common method bias, and reliability analyses were conducted for all study variables. Confirmatory factor analysis (CFA) was performed in Mplus to examine construct validity. After that, we computed descriptive statistics and bivariate correlations. To test the hypotheses, we ran hierarchical regression analyses and PROCESS analyses. We used PROCESS Model 4 to examine the mediating role of academic impostor syndrome and Model 7 to test the moderated mediation effect. We estimated indirect effects using bias-corrected bootstrapping with 5000 resamples. A 95% confidence interval (CI) that did not include zero was taken to indicate statistical significance.

## 4. Results

### 4.1. Preliminary Analyses

Preliminary analyses examined common method bias, measurement validity, and the assumptions for regression-based analyses. Harman’s single-factor test was used as a supplementary diagnostic for common method bias. The first unrotated factor accounted for 25.23% of the total variance, below the commonly used 40% threshold (Podsakoff et al., 2003). Given the limitations of this test, common method concerns were also addressed procedurally through the two-wave design, temporal separation between predictors and outcome-related variables, anonymous responses, and instructions emphasizing voluntary participation and confidentiality.

Confirmatory factor analysis was conducted to examine the distinctiveness and validity of the focal constructs. Academic engagement is commonly conceptualized as a multidimensional construct comprising behavioral, emotional, and cognitive components (Fredricks et al., 2004; Maroco et al., 2016), and the USEI has demonstrated cross-cultural validity (Assunção et al., 2020). Accordingly, academic engagement was modeled as a second-order latent factor indicated by these three first-order dimensions, while the remaining constructs were modeled as first-order latent factors with their respective items as indicators. The measurement model showed good fit to the data: χ^2^(616) = 1029.70, χ^2^/df = 1.672, RMSEA = 0.037, 90% CI [0.033, 0.041], CFI = 0.961, TLI = 0.957, SRMR = 0.047. All first-order standardized factor loadings were statistically significant, ranging from 0.569 to 0.902. The second-order loadings of academic engagement on behavioral engagement (*β* = 0.49), emotional engagement (*β* = 0.56), and cognitive engagement (*β* = 0.39) were also statistically significant (*p* < 0.001).

As shown in Table 2, composite reliability (CR) values ranged from 0.818 to 0.934, exceeding the recommended threshold of 0.70. Average variance extracted (AVE) values ranged from 0.534 to 0.675, exceeding the recommended threshold of 0.50 (Fornell & Larcker, 1981; Hair et al., 2019). These results supported adequate convergent validity. Discriminant validity was further supported because the square root of AVE for each construct exceeded its correlations with the other constructs (Fornell & Larcker, 1981).

The assumptions for regression-based analyses were also examined. Skewness values ranged from −0.407 to 0.255, and kurtosis values ranged from −1.326 to −0.712, all within the acceptable thresholds recommended by Kline (2016; |skewness| < 3, |kurtosis| < 10), indicating no severe deviations from univariate normality. Across both regression models predicting academic impostor syndrome and academic engagement, tolerance values ranged from 0.551 to 0.981, and VIF values ranged from 1.019 to 1.816, well below the commonly cited cutoffs of tolerance < 0.10 and VIF > 10 (Hair et al., 2019), indicating no serious multicollinearity. Histograms of standardized residuals approximated a normal distribution, and normal P-P plots showed that residuals fell closely along the diagonal reference line. Scatterplots of standardized residuals against standardized predicted values for both models showed a roughly random distribution of points around zero with no clear funnel-shaped or curvilinear patterns, supporting the assumptions of residual normality, linearity, and homoscedasticity for the planned regression and PROCESS analyses.

### 4.2. Descriptive Statistics and Correlation Analysis

Table 3 presents the means, standard deviations, and Pearson correlation coefficients for all study variables. Notably, there was no significant correlation between students’ AI use frequency and academic impostor syndrome (*r* = 0.06, *p* = 0.10) or academic engagement (*r* = −0.06, *p* = 0.22). This pattern indicates that AI use frequency alone may not adequately account for students’ psychological and academic outcomes, highlighting the value of distinguishing between distinct forms of AI use. In contrast, the two AI use styles exhibited distinct correlation patterns. Reflective AI use was negatively correlated with impostor syndrome (*r* = −0.24, *p* < 0.001), while thoughtless AI use was positively correlated with it (*r* = 0.30, *p* < 0.001). Furthermore, academic impostor syndrome showed a strong negative association with academic engagement (*r* = −0.41, *p* < 0.001).

### 4.3. Mediation Analysis of Academic Impostor Syndrome

We first conducted hierarchical regression to examine the direct paths (see Table 4). After controlling demographic factors and AI use frequency, reflective AI use negatively predicted academic impostor syndrome (*β* = −0.22, *p* < 0.001), supporting H1a. Conversely, thoughtless AI use positively predicted impostor syndrome (*β* = 0.28, *p* < 0.001), supporting H1b. In the final step of the regression (M5), academic impostor syndrome was a significant negative predictor of academic engagement (*β* = −0.37, *p* < 0.001).

Mediation was further verified using the PROCESS macro (Model 4) with 5000 bootstrap resamples. As shown in Table 5, the indirect effect of reflective AI use on engagement through impostor syndrome was 0.071, 95% CI [0.041, 0.105]. The indirect effect of thoughtless AI use was −0.097, 95% CI [−0.138, −0.062]. Since neither confidence interval included zero, it indicates that impostor syndrome mediates the association between AI use styles and engagement. These findings supported H2a and H2b.

### 4.4. Moderation and Moderated Mediation Analysis

We examined whether AI policy clarity (APC) moderated the first-stage associations between AI use styles and academic impostor syndrome using hierarchical regression analysis (Table 6). The two interaction terms were entered into separate models to test the moderation effects of reflective and thoughtless AI use. The interaction between reflective use and APC was significant (*β* = −0.26, *p* < 0.001). The interaction between thoughtless AI use and APC was also significant (*β* = 0.20, *p* < 0.001).

**Figure 2:**
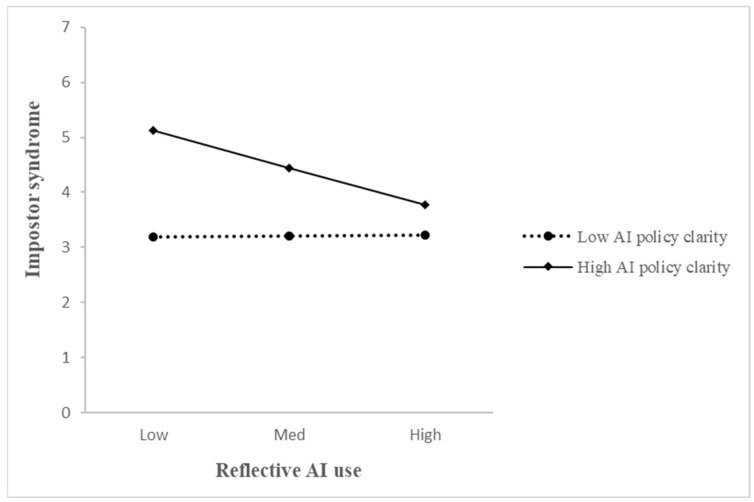
Interaction of reflective AI use and AI policy clarity on impostor syndrome.

**Figure 3:**
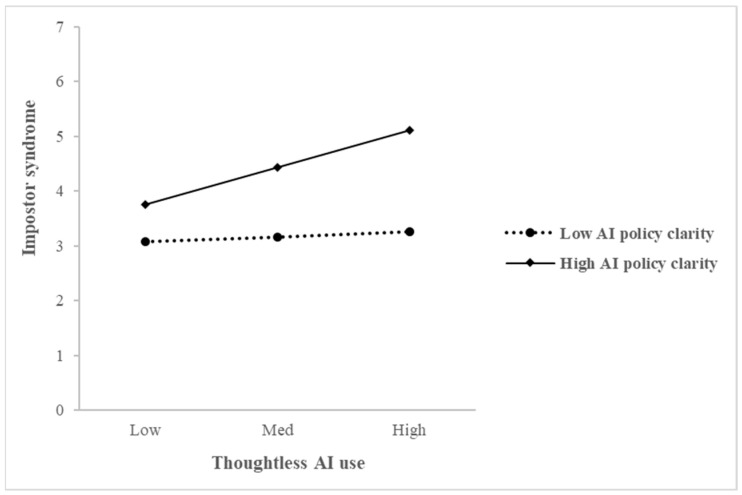
Interaction of thoughtless AI use and AI policy clarity on impostor syndrome.

Simple slope tests, as illustrated in Figure 2 and Figure 3, further clarified these moderation patterns. Reflective AI use was not significantly associated with academic impostor syndrome when APC was low (conditional effect = 0.01, *p* = 0.81), but was significantly and negatively associated with academic impostor syndrome when APC was high (conditional effect = −0.59, *p* < 0.001). Conversely, thoughtless AI use was not significantly associated with academic impostor syndrome when APC was low (conditional effect = 0.08, *p* = 0.19), but was significantly and positively associated with academic impostor syndrome when APC was high (conditional effect = 0.61, *p* < 0.001). These results suggest that policy clarity amplified the divergent associations of reflective and thoughtless AI use with impostor feelings, supporting H3a and H3b.

Finally, we tested the moderated mediation hypothesis using PROCESS Model 7 with 5000 bootstrap samples (Table 7). The index of moderated mediation was significant for the reflective AI use path, Index = 0.071, 95% CI [0.048, 0.099], and for the thoughtless AI use path, Index = −0.066, 95% CI [−0.092, −0.043]. Because both confidence intervals excluded zero, these results supported H4a and H4b, indicating that AI policy clarity moderated the indirect associations between AI use styles and academic engagement via academic impostor syndrome.

## 5. Discussion

### 5.1. AI Interaction Styles Versus Usage Frequency

Recent systematic reviews suggest that GenAI has become a consequential part of students’ everyday academic work (Wu et al., 2025). However, much of the literature to date examines AI use through the lens of broad adoption trends or overall intensity of use (Batista et al., 2024). Our results show that such a framework may be too simple to capture the actual links between AI use and students’ academic experiences. In the present data, the frequency of AI use showed no significant association with either psychological or academic outcomes. Rather, the nature of students’ interaction with AI turned out to be more important. This result supports the view that understanding AI reliance in educational contexts should go beyond focusing purely on quantity (Heung & Chiu, 2025).

These findings may also help explain inconsistencies in previous research. For example, Almohammadi et al. (2025) reported high rates of AI use among graduate students, but they did not find a significant link to impostor feelings. One plausible explanation is that treating AI use as a single, undifferentiated construct may mask meaningful variation in how students use AI in practice. When the quality of AI use is disentangled from frequency, we find a more fine-grained pattern that is broadly consistent with the Automation–Augmentation Paradox (Raisch & Krakowski, 2021). In line with the agency-centered framework developed by Yang et al. (2024), the results do not suggest that AI use per se produces academic impostor syndrome. Instead, what matters is whether students engage in thoughtless AI use that simply substitutes active cognitive judgment with a heuristic shortcut, which may then feed into feelings of inauthenticity. These insights have important implications for understanding how AI relates to students’ academic experiences. At the same time, such interpretations should be viewed with caution. Although this study identified divergent patterns linked to the two interaction styles, questions regarding boundary conditions and cross-context generalizability remain open.

### 5.2. Academic Impostor Syndrome as a Mediating Mechanism

Our results suggest that academic impostor syndrome may help account for the observed associations between different AI interaction styles and student academic engagement. Our findings therefore indicate that the effects of AI use are not just about learning efficiency, but also about whether students perceive their academic performance as genuinely reflecting their own competence and retain a sense of ownership over their academic work (Batista-Toledo & Gavilan, 2026; Perdana et al., 2026). In our study, reflective AI use was associated with lower levels of impostor feelings, which is in line with the idea that deliberate evaluation of AI outputs may support students in maintaining a strong sense of personal agency. When students engage with AI-generated content in a reflective and evaluative manner, they are more likely to feel their academic achievements are truly reflective of their own ability (Yang et al., 2024). By contrast, our results also support the idea that more automated or thoughtless use of AI may give rise to feelings of inauthenticity. Our focus on more automation-oriented use can be viewed as a form of cognitive outsourcing associated with a weaker perceived link between effort and academic outcomes (Domingo, 2025; Tsao, 2025). In such cases, students may be more inclined to perceive their achievements as unearned and to attribute success more to AI support than to their own competence. This may co-occur with fears of being exposed as incompetent or unworthy (Y. Wang & Li, 2023), which is related to the psychological resources needed for sustained academic engagement (Heung & Chiu, 2025). Collectively, these results suggest that when universities integrate AI into learning, supporting students’ sense of intellectual legitimacy may be as important as providing technical skills training.

### 5.3. Policy Clarity as a Contextual Amplifier

One notable finding of this study is that AI policy clarity appears to amplify students’ self-evaluative responses to different AI interaction styles. While prior research has emphasized the importance of policy clarity in shaping student attitudes (Tang et al., 2025), the present findings further highlight its dual role as a contextual amplifier. From a social information processing perspective, institutional policies provide environmental cues that students use to interpret the legitimacy and meaning of their academic behavior (Chan, 2023; Tsao, 2025). When policy boundaries are clear, reflective AI use may be more readily understood as a legitimate and responsible form of tool-supported learning. Under these conditions, students may experience less uncertainty about whether their AI-assisted work is academically acceptable, which may in turn make it easier to experience such performance as genuinely reflecting their own competence (Batista-Toledo & Gavilan, 2026; Perdana et al., 2026; Tsao, 2025; Yang et al., 2024). On the other hand, our results also suggest that policy clarity may make the impostor-related implications of thoughtless AI use more salient. If the parameters of acceptable AI use are clearly spelled out, uncritical use of AI tools may become more conspicuous to students as being at odds with legitimate academic engagement (Vassallo, 2026). Perceived misalignment with institutional expectations may be linked to stronger self-doubt and a weaker sense that one has fully earned one’s academic performance (Chan, 2023; Vassallo, 2026).

Beyond this moderating role, our findings also reveal a direct positive association between AI policy clarity and academic impostor syndrome, which deserves careful interpretation. Clearer policies may reduce ambiguity, but they may also make the normative boundaries of AI-supported academic work more salient. When students become more aware of what counts as acceptable, questionable, or excessive AI use, they may engage in stronger self-monitoring of their academic authorship and legitimacy. Recent work on AI policy and academic integrity suggests that when policy clarity is achieved primarily through detection-oriented or highly rule-based approaches, it may foster self-surveillance and anxiety, especially when students worry about being misidentified as engaging in misconduct (Tsao, 2025). Related research on AI guilt also indicates that students may experience moral discomfort when GenAI use appears to conflict with academic values of authenticity, individual effort, and intellectual ownership (Qu & Wang, 2025). In such contexts, students’ attention may be directed more toward demonstrating compliance than toward learning and authorship, which could weaken their sense of ownership over AI-assisted work (Batista-Toledo & Gavilan, 2026; Tsao, 2025). From a social information processing perspective, policy clarity may therefore function as an ambivalent contextual cue: it clarifies institutional expectations, but it may also heighten students’ sensitivity to whether their AI-assisted achievements genuinely reflect their own competence, effort, and authorship (Chan, 2023; Tang et al., 2025).

Overall, these findings suggest that AI policy clarity plays a dual role in students’ AI-assisted academic work. Its positive direct association with academic impostor syndrome suggests that clearer policy cues may heighten students’ awareness of academic legitimacy, authorship boundaries, and the authenticity of AI-assisted achievement. At the same time, its moderating role suggests that policy clarity may amplify the psychological meaning of different AI use styles. Specifically, it was associated with a stronger lower-impostor pattern for reflective AI use and a stronger higher-impostor pattern for thoughtless AI use. Policy clarity should therefore be understood neither as a purely protective factor nor as a purely risk-inducing condition, but as an ambivalent contextual cue that makes the self-evaluative implications of AI use more salient.

### 5.4. Theoretical Contributions

This study offers three distinct theoretical contributions to the literature on generative AI integration in higher education:

First, the study shifts theoretical attention from the quantity of GenAI use to the qualitative style of human–AI interaction. Most prior work treats AI use as a relatively uniform construct and focuses primarily on the prevalence of AI adoption or the overall frequency of AI use (Batista et al., 2024; Wu et al., 2025). Such a frequency-based framing has limited capacity to explain why similar levels of AI exposure can lead to divergent psychological and academic outcomes. By conceptualizing and empirically distinguishing reflective AI use from thoughtless AI use, the present research introduces a style-based perspective on human–AI interaction, reframing the central question from “how much students use AI” to “how students cognitively and metacognitively engage with AI.” This perspective offers a more nuanced explanation of why GenAI use may be associated with different self-evaluative experiences and levels of academic engagement.

Second, this study contributes to a more nuanced understanding of competence in human–AI collaborative learning. In traditional academic settings, task performance often provides relatively direct feedback about students’ ability. In GenAI-supported academic work, however, this feedback becomes more ambiguous because successful task completion may reflect both students’ own effort and judgment and the support provided by AI. Students may therefore feel confident in producing acceptable outcomes while remaining uncertain about how much of those outcomes can be attributed to their own competence. By examining academic impostor syndrome as the mediating variable, this study addresses this attributional ambiguity more directly than general measures of academic self-efficacy or task-completion confidence. It asks whether AI-assisted achievement is experienced as authentic, deserved, and attributable to students’ own effort and judgment. In this sense, the findings extend AI-assisted learning research from an outcome-oriented view of performance and perceived capability to a process-attribution view of competence in human–AI collaboration. What matters, therefore, is not AI use itself, but whether students experience AI-supported performance as genuinely their own (Batista-Toledo & Gavilan, 2026; Perdana et al., 2026; Y. Wang & Li, 2023; Zhang & Xu, 2025).

Third, drawing on Social Information Processing Theory, this study enriches the research on contextual factors in AI-assisted learning by finding that AI policy clarity is an important boundary condition that is associated with how students experience the psychological meaning and academic behaviors associated with AI use. Our findings show that policy clarity is linked to how students construe the meaning and academic legitimation of their AI-supported work. In this way, the institutional context not only regulates AI use externally but also appears to relate to how students think for themselves when using AI for their academic work. This moves beyond viewing institutional policy as a simple regulatory tool (Chan, 2023). Instead, the results suggest that policy clarity may operate as a salient normative cue linked to how students interpret their own behavior (Tang et al., 2025; Tsao, 2025). More specifically, clear policies may provide a more stable basis for reflective users to regard their AI-assisted work as legitimate, while making ethical boundaries more salient for students who rely on AI in a more thoughtless way (Vassallo, 2026). In this respect, the institutional environment may relate not only to whether students feel constrained in their use of AI, but also to the self-evaluative consequences attached to that use.

### 5.5. Practical Implications

#### 5.5.1. Developing Reflective AI Use Literacy

Rather than viewing AI as a neutral tool, students should be encouraged to develop reflective AI use literacy, which involves the metacognitive regulation of their AI interaction styles. This means learning to recognize, monitor, and regulate the qualitative differences between reflective use and thoughtless use (Hou et al., 2025a, 2025b). Recent work on self-regulation for AI-based learning further supports this point. Yurt (2025) suggests that effective AI-assisted learning depends on students’ ability to regulate their motivation, cognitive and metacognitive strategies, task management, resource use, and technological adaptation. In practice, students need to set learning goals before using AI, monitor whether AI is supporting or replacing their own thinking, evaluate the quality of AI-generated content, and adjust their AI use when it appears to diminish active engagement. This involves asking whether AI is functioning as a cognitive scaffold that supports thinking, judgment, and revision, or as a substitute for students’ own cognitive effort (Marín Díaz, 2025; Perdana et al., 2026). Practical tools such as prompt logs, revision memos, and reflective checklists can support this process by requiring students to document how AI-generated outputs were evaluated, revised, and integrated into their own work. By cultivating this form of reflective AI use literacy, institutions can help students understand that over-reliance on AI may weaken their sense of competence, authenticity and psychological ownership, while more regulated and reflective use may support students in attributing academic success to their own agency (Batista-Toledo & Gavilan, 2026; Y. Wang & Li, 2023).

#### 5.5.2. Educator Modeling and Coaching Role

Building on the development of students’ self-awareness of AI interaction patterns, instructors can move beyond traditional content delivery and take on a more intentional coaching role in AI-integrated learning settings (Rahimi & Sevilla-Pavón, 2024; S. J. Wang & Huang, 2026). One concrete approach is to model constructive human–AI collaborative practices in classroom activities (Chang & Kidman, 2023; Yang et al., 2024). This aligns with recent evidence from Chinese higher education, which shows that guided exposure, teacher modeling, and explicit discussion of AI limitations can help students identify legitimate learning uses of ChatGPT and develop more responsible routines (Day, 2026). Such guidance also depends on educators’ own readiness and institutional support; research on Chinese university lecturers suggests that AI literacy, professional development, and perceived support are important for responsible GenAI adoption (Luo & Day, 2026). For example, instructors can demonstrate how to maintain cognitive control by refining prompts, critically assessing AI outputs, verifying information, and revising student-generated work. These strategies help students learn to treat AI as a support tool rather than a replacement for their own thinking (Perdana et al., 2026). Such modeling can help students develop epistemic vigilance, which is important for maintaining academic authorship and confidence that their work reflects their own cognitive effort (Batista-Toledo & Gavilan, 2026; Perdana et al., 2026).

#### 5.5.3. Redesigning Tasks for Process-Oriented Collaboration

At the curriculum level, task design can be improved by moving from unstructured to structured (and AI-supported) tasks that still require a certain degree of active cognitive reasoning from students (Batista-Toledo & Gavilan, 2026). One possible strategy is to implement a Generate–Reflect–Calibrate cycle, meaning that students submit not only their final academic work but also process-based records documenting how they integrated AI into their thinking and revision (Xu et al., 2025). For instance, these records could include logs of their interactions with AI, the revision history of their drafts, or reflective memos on the changes they made using AI (Tsao, 2025). This form of assessment helps maintain students’ active role in the learning process and reinforces the perceived link between students’ personal effort and their academic outcomes (Batista-Toledo & Gavilan, 2026; Domingo, 2025).

#### 5.5.4. Enhancing Institutional Guidance and Psychological Support

At the institutional level, the wider institutional context should create a clear and supportive environment that eases the interpretive burden students face when using AI for academic work (Chan, 2023; Tang et al., 2025). Universities can go beyond a strictly demanding stance and provide concrete examples of acceptable and unacceptable AI use, along with active, educational ethical guidance (Tsao, 2025; Vassallo, 2026). On campus, student support resources should also be more responsive to concerns about the authenticity of academic work and the uncertainty that can arise from over-reliance on AI (Almohammadi et al., 2025; Vassallo, 2026). The combination of academic skills guidance and psychological counseling could be particularly useful for students with a strong dependence on AI or weak perceived competence, as it could support the restoration of academic self-efficacy and the scholarly self in an AI-abundant learning environment (Y. Wang & Li, 2023).

### 5.6. Limitations and Future Research Directions

This study has several limitations that merit recognition.

First, although the two-wave design reduced some concerns related to common method bias and temporal ambiguity, causal inference remains limited. Academic impostor syndrome and academic engagement were both measured at Time 2, which prevents strong conclusions about their temporal ordering. Therefore, the mediation findings should be interpreted as theoretically guided indirect associations rather than definitive causal mechanisms. Future research should use three-wave longitudinal designs, cross-lagged models, experimental designs, or behavioral log data to examine causal and reciprocal relationships among AI use styles, impostor feelings, and academic engagement.

Second, the results are based on self-reported measures that could be subject to social desirability bias or differences in how students perceive reflective versus thoughtless AI use. Future studies could incorporate more objective behavioral measures, such as AI interaction logs, prompt revisions, or instructor ratings, to obtain a more fine-grained, triangulated view of human–AI collaboration processes.

Third, this study adopted a self-focused theoretical perspective by using academic impostor syndrome as the core mediating mechanism linking AI interaction styles to academic engagement. While this aligns with our interest in self-perception and authenticity concerns in AI-assisted learning, the underlying psychological mechanisms are likely more multifaceted. In the future, it would be worthwhile to test other mediating mechanisms from different theoretical perspectives, for instance, cognitive factors (e.g., cognitive load, metacognitive regulation), motivational factors (e.g., intrinsic motivation, achievement goal orientations), and/or emotional factors (e.g., learning anxiety, academic passion). Moreover, although this study only took academic engagement as the outcome variable, future work could consider a broader range of outcomes, such as objective academic performance, critical thinking skills, creativity, or long-term learning trajectories. Besides AI policy clarity, other contextual factors may also matter, for instance, instructor support, peer collaboration norms, task complexity, and disciplinary culture.

Finally, the practical implications discussed herein are primarily conceptual and will require further empirical validation. Future studies could test the intervention effect of training sessions focused on guiding students to reflect on their AI use styles and examine whether these interventions can enhance students’ sense of academic authenticity, perceived competence, and academic engagement.

## 6. Conclusions

This study examined how different styles of GenAI use in core academic tasks are related to students’ academic impostor syndrome and academic engagement. Using a two-wave survey of Chinese university students, the findings suggest that reflective and thoughtless AI use show distinct psychological and academic patterns. Reflective AI use was associated with lower academic impostor syndrome and indirectly with higher academic engagement, whereas thoughtless AI use showed the opposite pattern. Overall, the study offers empirical evidence for distinguishing reflective and thoughtless AI use as qualitatively different patterns of human–AI collaboration. It also suggests that the qualitative style of AI use may be more informative than AI use frequency alone.

This study also highlights academic impostor syndrome as a meaningful self-evaluative mechanism in GenAI-supported academic work. In human–AI collaboration, students may not only consider whether they can complete a task, but also whether the outcome genuinely reflects their own effort, judgment, and competence. By focusing on this process of competence attribution, the study extends AI-assisted learning research beyond task completion, performance outcomes, perceived capability, or general technology adoption, and links these self-evaluative processes to students’ sustained academic engagement.

In addition, the findings suggest that perceived AI policy clarity plays an ambivalent contextual role. Clearer institutional guidance may help students interpret reflective AI use as a legitimate form of academic support, but it may also make questions of authorship, legitimacy, and acceptable use more salient. Thus, policy clarity should not be understood simply as a protective factor. Rather, it may amplify the self-evaluative implications associated with different AI use styles. In this sense, higher policy clarity appeared to strengthen both the lower-impostor pattern associated with reflective AI use and the higher-impostor pattern associated with thoughtless AI use.

Taken together, this study shifts attention from how often students use AI to how they use it, and from whether AI helps students complete tasks to how students interpret their own competence and sustain engagement in AI-supported academic work. For higher education institutions, the findings suggest the need to cultivate reflective AI literacy, support students’ academic authorship, and provide clear but psychologically sensitive policy guidance. Because the mediator and outcome were measured at the same time point, the mediation findings should be interpreted as theoretically guided rather than definitively causal. Future research should use stronger longitudinal or experimental designs to further examine the directionality and boundary conditions of these relationships.

## Figures and Tables

**Table 1:** Basic information of the sample (*N* = 478).

Variable	Category	Frequency	Percentage (%)
Gender	Male	199	41.6
	Female	279	58.4
Academic year	Freshman	139	29.1
	Sophomore	94	19.7
	Junior	139	29.1
	Senior	106	22.2
Discipline	Humanities and Social Sciences	93	19.5
	Science	130	27.2
	Engineering	133	27.8
	Agriculture	76	15.9
	Medicine	46	9.6
Academic Performance	Top 10%	47	9.8
	Top 10–25%	159	33.3
	Top 26–50%	145	30.3
	51–75%	109	22.8
	Bottom 25%	18	3.8

**Table 2:** Composite Reliability, AVE, and Discriminant Validity.

Construct	CR	AVE	√AVE	1	2	3	4	5	6	7
1. Thoughtless AI use	0.818	0.534	**0.731**	-						
2. Reflective AI use	0.841	0.571	**0.756**	−0.018						
3. AI policy clarity	0.878	0.643	**0.802**	0.110	0.028	-				
4. Academic Impostor Syndrome	0.934	0.586	**0.765**	0.334	−0.271	0.482	-			
5. Behavioral Engagement	0.898	0.639	**0.799**	−0.062	0.155	−0.066	−0.343	-		
6. Emotional Engagement	0.912	0.675	**0.821**	−0.071	0.180	−0.077	−0.396	0.273	-	
7. Cognitive Engagement	0.911	0.674	**0.821**	−0.050	0.125	−0.053	−0.275	0.190	0.219	-

*Note.* Bold diagonal values represent the square root of AVE. Off-diagonal values are latent factor correlations from the CFA. CR = composite reliability; AVE = average variance extracted.

**Table 3:** Descriptive statistics and correlational analysis results (*N* = 478).

Variables	M	SD	1	2	3	4	5	6	7	8	9	10
1 Gender	0.58	0.49	1									
2. AY	2.44	1.13	−0.10 *	1								
3. Major	2.69	1.23	−0.01	−0.04	1							
4. AP	3.23	1.03	0.05	−0.10 *	0.06	1						
5. Freq	3.58	1.36	−0.08	−0.05	−0.05	0.09	1					
6. RAU	3.28	1.13	0.05	−0.03	−0.02	0.20 ***	−0.05	1				
7. TAU	2.88	1.10	0.06	0.10 *	−0.08	−0.02	0.06	−0.03	1			
8. APC	3.14	1.13	0.08	0.03	−0.02	0.14 **	0.120 **	0.02	0.09	1		
9. AIS	3.82	1.36	0.04	0.07	−0.13 **	−0.20 **	0.06	−0.24 ***	0.30 ***	0.45 ***	1	
10. AE	3.13	0.97	−0.03	−0.04	0.09	0.20 ***	−0.06	0.20 ***	−0.09	−0.08	−0.41 ***	1

Note: AY = Academic year; AP = Academic performance; Freq = Frequency; RAU = Reflective AI use; TAU = Thoughtless AI use; APC = AI policy clarity; AIS = Academic impostor syndrome; AE = Academic engagement. * *p* < 0.05; ** *p* < 0.01; *** *p* < 0.001. Control variable coding is described in the Section 3.2.

**Table 4:** Regression analysis of mediating effects.

	Academic Impostor Syndrome						Academic Engagement			
	M1		M2		M3		M4		M5	
	*B (SE)*	*β*	*B (SE)*	*β*	*B (SE)*	*β*	*B (SE)*	*β*	*B (SE)*	*β*
Constant	4.10 *** (0.34)		4.00 *** (0.37)		2.66 *** (0.24)		2.41 *** (0.28)		3.47 *** (0.29)	
Gender	0.17 (0.13)	0.06	0.14 (0.12)	0.05	−0.08 (0.09)	−0.04	−0.09 (0.09)	−0.04	−0.05 (0.08)	−0.03
Academic year	0.08 (0.06)	0.07	0.04 (0.05)	0.04	−0.02 (0.04)	−0.03	−0.01 (0.04)	−0.02	−0.004 (0.04)	−0.004
Major	−0.13 * (0.05)	−0.12 *	−0.12 * (0.05)	−0.11 *	0.06 (0.04)	0.07	0.06 (0.04)	0.07	0.03 (0.03)	0.03
Academic performance	−0.15 * (0.06)	−0.11 *	−0.09 (0.06)	−0.07	0.19 *** (0.04)	0.20 ***	0.16 *** (0.04)	0.17 ***	0.14 ** (0.04)	0.14 **
AI use frequency	0.07 (0.05)	0.07	0.04 (0.04)	0.04	−0.05 (0.03)	−0.07	−0.04 (0.03)	−0.06	−0.03 (0.03)	−0.05
Reflective AI use			−0.27 *** (0.05)	−0.22 ***			0.14 *** (0.04)	0.16 ***	0.07 (0.04)	0.08
Thoughtless AI use			0.34 *** (0.05)	0.28 ***			−0.06 (0.04)	−0.07	0.03 (0.04)	0.04
Impostor syndrome									−0.27 *** (0.03)	−0.37 ***
*R* ^2^	0.040		0.165		0.053		0.083		0.199	
Δ*R*^2^	0.040		0.125		0.053		0.030		0.116	
*F*	*F*(5, 472) = 3.97 **		*F*(7, 470) = 13.30 ***		*F*(5, 472) = 5.32 ***		*F*(7, 470) = 6.09 ***		*F*(8, 469) = 14.58 ***	
Δ*F*	Δ*F*(5, 472) = 3.97 **		Δ*F*(2, 470) = 35.17 ***		Δ*F*(5, 472) = 5.32 ***		Δ*F*(2, 470) = 7.65 **		Δ*F*(1, 469) = 67.88 ***	

Note: * *p* < 0.05; ** *p* < 0.01; *** *p* < 0.001. Control variable coding is described in the Section 3.2.

**Table 5:** Summary of indirect effects.

Paths	Estimates	SE	95% CI
Reflective AI use → Impostor syndrome → Academic engagement			
Indirect effect	0.071	0.016	[0.041, 0.105]
Direct effect	0.069	0.037	[−0.004, 0.143]
Thoughtless AI use → Impostor syndrome → Academic engagement			
Indirect effect	−0.097	0.019	[−0.138, −0.062]
Direct effect	0.036	0.039	[−0.039, 0.112]

Notes: N = 478. Bootstrap samples = 5000. CI = confidence interval.

**Table 6:** Regression analysis for moderating effects.

	Academic Impostor Syndrome							
	M1		M6		M7		M8	
	*B (SE)*	*β*	*B (SE)*	*β*	*B (SE)*	*β*	*B (SE)*	*β*
Constant	4.10 *** (0.34)		4.63 *** (0.28)		4.44 *** (0.27)		4.60 *** (0.28)	
Gender	0.17 (0.13)	0.06	0.04 (0.10)	0.02	0.09 (0.10)	0.03	0.02 (0.10)	0.01
Academic year	0.08 (0.06)	0.07	0.02 (0.05)	0.02	−0.001 (0.04)	−0.001	0.02 (0.04)	0.02
Major	−0.13 * (0.05)	−0.12 *	−0.11 * (0.04)	−0.10 *	−0.09 * (0.04)	−0.08 *	−0.10 * (0.04)	−0.09 *
Academic performance	−0.15 * (0.06)	−0.11 *	−0.17 ** (0.05)	−0.13 **	−0.12 * (0.05)	−0.09 *	−0.16 ** (0.05)	−0.12 **
AI use frequency	0.07 (0.05)	0.07	−0.01 (0.04)	−0.01	−0.005 (0.04)	−0.01	−0.02 (0.04)	−0.02
Reflective AI use			−0.27 *** (0.05)	−0.22 ***	−0.28 *** (0.04)	−0.24 ***	−0.26 *** (0.04)	−0.22 ***
Thoughtless AI use			0.30 *** (0.05)	0.25 ***	0.30 *** (0.04)	0.24 ***	0.34 *** (0.05)	0.28 ***
AI policy clarity			0.54 *** (0.05)	0.44 ***	0.53 *** (0.04)	0.44 ***	0.56 *** (0.04)	0.47 ***
RAU × APC					−0.27 *** (0.04)	−0.26 ***		
TAU × APC							0.23 *** (0.04)	0.20 ***
*R* ^2^	0.040		0.353		0.416		0.393	
Δ*R*^2^	0.040		0.312		0.064		0.040	
*F*	*F*(5, 472) = 3.97 **		*F*(8, 469) = 31.95 ***		*F*(9, 468) = 37.12 ***		*F*(9, 468) = 33.60 ***	
Δ*F*	Δ*F*(5, 472) = 3.97 **		Δ*F*(3, 469) = 75.46 ***		Δ*F*(1, 468) = 51.13 ***		Δ*F*(1, 468) = 30.63 ***	

Note: RAU = Reflective AI use; TAU = Thoughtless AI use; APC = AI policy clarity. * *p* < 0.05; ** *p* < 0.01; *** *p* < 0.001. Control variable coding is described in the Section 3.2.

**Table 7:** Moderated mediation results.

Independent Variable	Levels of AI Policy Clarity	Estimates	SE	95% CI	Index of Moderated Mediation	
					Index	95% CI
Reflective AI use	Low (−1 SD)	−0.005	0.014	[−0.033, 0.020]	0.071	[0.048, 0.099]
	High (+1 SD)	0.155	0.025	[0.109, 0.209]		
	Intergroup differences	0.157	0.028	[0.108, 0.217]		
Thoughtless AI use	Low (−1 SD)	−0.023	0.015	[−0.053, 0.007]	−0.066	[−0.092, −0.043]
	High (+1 SD)	−0.171	0.028	[−0.231, −0.119]		
	Intergroup differences	−0.148	0.029	[−0.208, −0.097]		

Notes: N = 478. Bootstrap samples = 5000. CI = confidence interval. Conditional indirect effects are reported at ±1 SD of AI policy clarity.

## Data Availability

The data presented in this study are available upon reasonable request from the corresponding author due to ethical and privacy restrictions related to informed consent and participant data confidentiality.

## Abbreviations

The following abbreviations are used in this manuscript:

RAU	Reflective AI use
TAU	Thoughtless AI use
APC	AI policy clarity
AIS	Academic impostor syndrome
AE	Academic engagement
